# A SLC16A1 Mutation in an Infant With Ketoacidosis and Neuroimaging Assessment: Expanding the Clinical Spectrum of MCT1 Deficiency

**DOI:** 10.3389/fped.2019.00299

**Published:** 2019-07-18

**Authors:** Sara Al-Khawaga, Jehan AlRayahi, Faiyaz Khan, Saras Saraswathi, Reem Hasnah, Basma Haris, Idris Mohammed, Essam M. Abdelalim, Khalid Hussain

**Affiliations:** ^1^College of Health and Life Sciences, Hamad Bin Khalifa University, Qatar Foundation, Education City, Doha, Qatar; ^2^Division of Endocrinology, Department of Pediatric Medicine, Sidra Medicine, Doha, Qatar; ^3^Diabetes Research Center, Qatar Biomedical Research Institute, Hamad Bin Khalifa University, Qatar Foundation, Doha, Qatar; ^4^Division of Neuroradiology, Diagnostic Imaging, Sidra Medicine, Doha, Qatar

**Keywords:** SLC16A1, MCT1, ketoacidosis, hypoglycemia, heterotopia, white matter disease, gray matter disease

## Abstract

The solute carrier family 16 member 1 (*SLC16A1*) gene encodes for monocarboxylate transporter 1 (MCT1) that mediates the movement of monocarboxylates, such as lactate and pyruvate across cell membranes. Inactivating recessive homozygous or heterozygous mutations in the *SLC16A1* gene were described in patients with recurrent ketoacidosis and hypoglycemia, a potentially lethal condition. In the brain where MCT1 is highly localized around axons and oligodendrocytes, glucose is the most crucial energy substrate while lactate is an alternative substrate. MCT1 mutation or reduced expression leads to neuronal loss due to axonal degeneration in an animal model. Herein, we describe a 28 months old female patient who presented with the first hypoglycemic attack associated with ketoacidosis starting at the age of 3 days old. Whole exome sequencing (WES) performed at 6 months of age revealed a c.218delG mutation in exon 3 in the *SLC16A1* gene. The variant is expected to result in loss of normal MCT1 function. Our patient is amongst the youngest presenting with MCT1 deficiency. A detailed neuroimaging assessment performed at 18 months of age revealed a complex white and gray matter disease, with heterotopia. The threshold of blood glucose to circumvent neurological sequelae cannot be set because it is patient-specific, nevertheless, neurodevelopmental follow up is recommended in this patient. Further functional studies will be required to understand the role of the MCT1 in key tissues such as the central nervous system (CNS), liver, muscle and ketone body metabolism. Our case suggests possible neurological sequelae that could be associated with MCT1 deficiency, an observation that could facilitate the initiation of appropriate neurodevelopmental follow up in such patients.

## Introduction

The main ketone bodies are acetoacetate, 3-hydroxybutyrate, and acetone, which act as the primary circulating energy source during fasting. Ketogenesis, a three-step enzymatic process, result in the formation of ketone bodies in the liver (1). Ketone bodies play a critical role in biologically active tissues where they are converted to acetyl-CoA, acting as an energy source during energy depletion (fasting and or prolonged exercise) (1). A significant proportion, ranging up to two-thirds, of the brain's energy demand is provided by ketone bodies following extended starvation (2). Ketoacidosis occurs when ketone formation exceeds that of their normal utilization, resulting in a pathological status. The clinical manifestations of genetic inborn errors of ketone utilization are similar to those seen in diabetic ketoacidosis. While glucose levels are normal to low in ketone body utilization disorders, a higher glucose level is seen in diabetic ketoacidosis (3). Symptoms are expected to be those seen in diabetic ketoacidosis and include vomiting, dehydration, Kussmaul breathing, and osmotic diuresis, which could ultimately advance to decreased consciousness and death (2).

Succinyl-CoA: 3-oxoacid CoA transferase (SCOT) (4) and mitochondrial acetoacetyl-CoA thiolase (ACAT1) (5) deficiencies, characterized by severe intermitten ketoacidosis evident in the neonatal periods, are among the originally identified genetic causes of recurrent ketoacidosis. Efficient ketolysis depends on the functioning of both SCOT and ACAT1, which are critical for the breakdown of ketone bodies into acetyl CoA, acting as a primary cellular energy source (6). More recently, inactivating mutations in Monocarboxylate Transporter 1 (MCT1), both homozygous and heterozygous, have been reported as a cause of profound ketoacidosis (6). MCT1, a transmembrane transporter encoded by solute carrier family 16 member 1 (*SLC16A1)* gene, transports monocarboxylates, including lactate and ketone bodies (7). Glycoprotein CD147, also called BSG, is required by MCT1 for proper subcellular expression (8, 9).

We report a variant in the *SLC16A1* gene encoding MCT1 in a neonate with recurrent acidosis, hypoglycemia, and neurological sequelae. Individuals with ketoacidosis provoked by fasting or infections have been reported with inactivating homozygous or heterozygous pathogenic variants in the SLC16A1 gene, however hypoglycemia was seen infrequently (6). The individuals with homozygous pathogenic variants presented at younger ages, had more profound ketoacidosis, and mild to moderate developmental delay (6). Treatment with intravenous glucose or dextrose results in blood gas profile improvement and clearance of metabolic acidosis. The heterozygous family members of patients with homozygous *SLC16A1* pathogenic variants did not report episodes of ketoacidosis, suggesting that heterozygous pathogenic variants result in ketoacidosis when combined with specific genetic and environmental factors (6).

## Case Presentation

We describe a 28 months old female patient born to a first-degree consanguineous parent. The patient was born at full term following a normal vaginal delivery with a birth weight of 3.6 Kg. Prenatal history was uneventful, with normal antenatal follow up and ultrasound screening performed at 20 weeks of gestation. The patient Apgar score was nine. At birth, the patient's vitals were within normal limits (Heart rate 130 beats per minute, respiratory rate 45 breaths per minute, systolic blood pressure 65 mm Hg, temperature 36.9°C). Neonatal examinations showed pink skin, with the patient resting symmetrically with the arms and legs in flexion, cried vigorously when stimulated, and moved all extremities equally. The red reflex assessment was normal with symmetry in both eyes, without opacities, white spots, or dark spots. There were no signs of dysmorphisms or congenital malformation. The neck showed full range of motion. There were no signs of neonatal infections or perinatal asphyxia. At three days of age, she presented with hyperthermia (temperature 37.8°C, HR: 140, RR: 42, BP: 90/45), hypoglycemia (2.8 mmol/L) and metabolic acidosis with normal lactic acid. This was managed with intravenous fluids, intravenous dextrose, and sodium chloride intravenously (IV). Full septic workup for group B streptococci, enteric gram-negative *Escherichia coli*, Listeria monocytogenes, gonococci, chlamydia, rubella, toxoplasma, cytomegalovirus (CMV), syphilis, and herpes simplex virus were negative. At presentation, a comprehensive metabolic screening including aminoacidopathy, organic aciduria, and urea cycle defects was normal. Serum lactate, pyruvate, amino acids were normal. Urine succenylacetone, orotic, and organic acids were also normal. Evaluation of galactosemia, glycogen storage disease, and mitochondria diseases were also negative. Galactose-1-phosphate uridyltransferase level was normal. Assessment for fatty acid oxidation defects, total and free carnitine levels, and acyl carnitine were normal. The patient's lipid profile is also within normal limits. Hormonal deficiency was also excluded (normal thyroid function tests, TSH, ACTH, growth hormone, and cortisol levels). Despite extensive investigations, no septic, metabolic, or endocrine causes of the metabolic acidosis or the hypoglycemia were established and the patient was discharged home. It was recommended that she should have frequent feeds. At 6 months (and also at 11, 13, and 14 months) of age, the patient continued to present with seizures, vomiting, hypoglycemia, and metabolic acidosis (Table 1). Comprehensive investigations performed at second presentation at 6 months of age were positive for high anion gap metabolic acidosis (21.05 mmol/L), hypoglycemia, and ketonuria (Table 1). A similar profile was also evident at 11, 13, and 14 months of age. Each acute episode of illness was managed with intravenous 0.9% saline, intravenous dextrose and intravenous sodium bicarbonate. The parents were advised not to fast the patient for long periods of time and feed frequently. The parents were also asked to look for signs of any intercurrent illness and check the patients blood glucose and seek medical help early.

**Table 1:** Biochemical profile during episodes of ketoacidosis.

**Investigation**	**Value**	**Reference**
Bicarbonate (mmol/L)	10	24–30
Sodium (mmol/L)	154	135–145
Potassium (mmol/L)	5.5	3.7–5.9
Chloride (mmol/L)	118	96–110
Calcium (mmol/L)	2.32	2.1–2.64
Albumin (gm/L)	49	28–44
Glucose (mmol/L)	2.8	3.3–5.5
**URINALYSIS**		
UA ketones	+++	
UA glucose	4+	
**BLOOD GAS PROFILE**		
PH arterial (Units)	7.0	7.35–7.45
pCO2 (mmHg)	25.5	35–48
Sodium (mmol/L)	152	135–145
Potassium (mmol/L)	2.8	3.5–5
Chloride (mmol/L)	122	96–110
Bicarbonate (mmHg)	6.7	13–29
Base excess arterial	−22.2	−3–2
Corrected anion gap for albumin (mmol/L)	21.05	High anion gap metabolic acidosis

*The investigation was carried on second presentation at 6 months of age. A similar profile was evident at 11, 13, and 14 months of age*.

**Figure 1:**
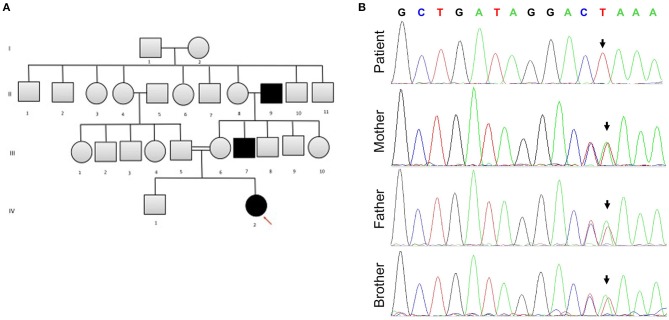
**(A)** The patient's family pedigree. The chart is significant for consanguinity and a similar phenotype observed in several family members. Arrow points to the affected patient. **(B)** Sanger sequencing for SLC16A1 mutation analysis. The patient is homozygous for *SLC16A1* c.218delG in Exon 3. Heterozygosity for c.218delG in Exon 3 of the SLC16A1 gene is displayed in the parents and sibling (brother). The Reverse Primer was used in the analysis. The colors represent bases: green denotes A, red T, black G, and blue C. Forward Primer, GAGGGAGCAGTTTCCTTTATATT; reverse primer, CTCACCTCCAATGACTCCAA.

Following the exclusion of metabolic disorders, and due to the significant family history for consanguinity in the parents (first-degree cousins) and a similar phenotype seen in two different generations in the maternal family (Figure 1A), genetic analysis in the family was recommended. At 6 months of age, Whole Exome Sequencing (WES) analysis was performed on the patient and her parents. Written informed consent forms were completed from all family members involved in the study. Genomic DNA of the patient and parents were isolated from peripheral blood samples for molecular genetic analysis. The extraction and purification was conducted according to the manufacturer's protocol (QIAamp DNA Blood Maxi Kit, Qiagen, Cat No.: 51194). Next-generation sequencing using an Illumina system was used to capture the exonic region and flanking splice junctions of the genome. All potentially pathogenic variants were confirmed using Capillary sequencing. Sequence and copy number alterations were reported according to the Human Genome Variation Society (HGVS) and International System for human Cytogenetic Nomenclature (ISCN) guideline, respectively. Sanger sequencing was used to confirm the mutation in the patient, both parents, and sibling. Applied Biosystems Sequencing Analysis Software v6.0 and Applied Biosystems SeqScape Software v3.0 were used for Sanger sequencing analysis. The patient has been shown to be homozygous for the c.218delG pathogenic variant in the *SLC16A1* Gene (p.Gly73ValfsX8 in exon 3 in the *SLC16A1* gene, rs1553208520) (Figure 1B). The patient's mother and father are heterozygous carriers for the c.218delG variant in the *SLC16A1* Gene (Figure 1B).

**Figure 2:**
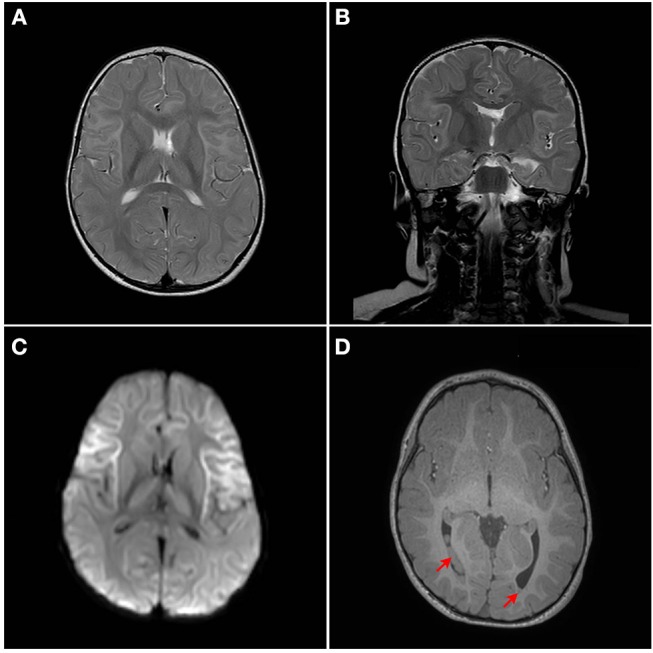
MRI Brain. Bilateral symmetrical abnormal T2/FLAIR hyperintense white matter signal changes with diffusion restriction are seen mainly involving the superficial subcortical U fibers with an anterior pre-dominance and involvement of the subinsular white matter. The corpus callosum, thalami, and basal ganglia are also involved. **(A)** Axial T2W1, **(B)** Coronal T2W1, **(C)** Axial DW1, and **(D)** Axial T1W1. Red arrows points to heterotopia on T1W1.

Although developmentally, at the age of 18 months, the patient has so far normal gross motor, fine motor, and language skills, assessment of neurological functions was initiated. The repeated episodes of seizure mandated an assessment of neurological sequelae, where a brain MRI was performed at the age of 18 months. MRI showed bilateral symmetrical abnormal T2/Fluid attenuated inversion recovery (FLAIR) hyperintense cortical/subcortical white matter signal involving the superficial subcortical U fibers of the frontal lobes, sub insular white matter, and the anterior temporal, parietal, and occipital lobes with corresponding significant diffusion restriction on Diffusion-weighted magnetic resonance imaging (DWI) (Figures 2A–D). Heterogeneous T2/FLAIR signal changes were also noted involving the basal ganglia and thalami with corresponding diffusion restriction. Diffusion restriction was also seen involving the rostrum and anterior body of the corpus callosum. In addition, multiple bilateral gray matter heterotopias were noted involving the posterior horns and trigones of the lateral ventricles bilaterally (Figures 2A–D). There is a signal change involving the cerebellar dentate nuclei. Age appropriate myelination without volume loss was also noted. MR spectroscopy appeared unremarkable with normal N-acetylaspartate (NAA), choline and creatine without a significant level of lactate.

Interestingly, our patient continued to demonstrate an increase in body weight and food seeking behavior. She has exhibited an increase in body weight, (above 97th percentile), a phenotype that could be attributed to MCT1 inhibition.

## Discussion

The *SLC16A1* gene encodes for MCT1 that mediates the movement of monocarboxylates, such as lactate and pyruvate across cell membranes (7). In the brain, lactate delivery to neuronal cells produced by astrocytes is controlled by monocarboxylate transporters (MCTs), and dysfunctional MCTs are associated with epilepsy (10, 11), cognitive defects (12), neurodegeneration (13, 14), and metabolic disorders (15). Recently, inactivating recessive homozygous or heterozygous (in exonic regions) mutations in the *SLC16A1* gene was described in patients with recurrent ketoacidosis and hypoglycemia (6). Other genetic causes of recurrent ketoacidosis are SCOT deficiency and ACAT1 deficiency, both of which are involved in ketolysis (2). Interestingly, heterozygous mutation in the SLC16A1 gene can also lead to clinical symptoms of MCT1 deficiency (16). It is possible that heterozygous mutation are subjected to nonsense mediated mRNA decay (NMD), where haploinsufficiency is a predicted *in vivo* mechanism responsible for the phenotypes associated with heterozygous nonsense alleles (17). The consequent lethal ketoacidosis, caused by the inadequate balance between hepatic ketone bodies production and their extrahepatic utilization, required urgent correction of the resulting electrolyte imbalance (3).

We report a mutation in the *SLC16A1* gene leading to recurrent ketoacidosis and hypoglycemia. The c.218delG variant occurs in exon 3 in the *SLC16A1* gene (NM_003051.3). The effect of the mutation at the protein level is difficult to predict, however, aberrant splicing could be one of the predicted mechanism for non-functional MCT1. The variant could also possibly causes a frameshift starting with codon Glycine 73, changes this amino acid to Valine residue, and creates a premature stop codon at position 8 of the new reading frame. Therefore, the variant seen in our patient is expected to result in loss of normal MCT1 protein function. The aberrant protein function is either attributed to MCT1 truncation or nonsense-mediated mRNA decay. Furthermore, mutations in the canonical acceptor and donor sites affect strongly conserved sequences that define exon-intron boundaries (18). Any variants in the canonical sequences might alter the interaction between pre-mRNA and proteins involved in the intron removal, where our reported mutation, occurring at the splice-acceptor site, is predicted to result in aberrant splicing (18). *In silico* analysis of the c.218delG variant of the *SLC16A1* gene is deleterious and is not detected in large population cohorts (19), therefore we interpret c.218delG as a pathogenic variant. Autosomal recessive inheritance is likely in this patient due to the homozygous presence of the variant and lack of *SLC16A1* phenotype in the parents.

**Figure 3:**
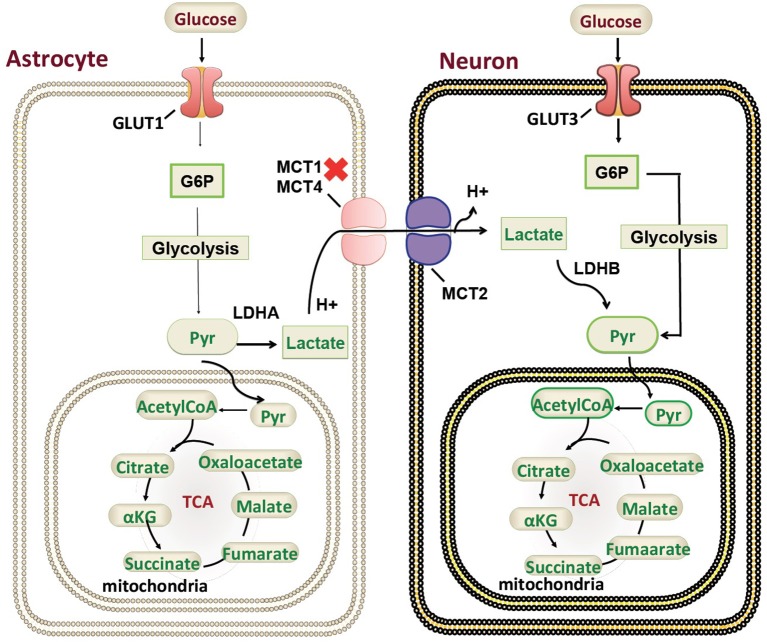
Monocarboxylate 1 transporter (MCT1) in the mammalian brain. A schematic representation of the genetic mutations and cellular localization of MCT1) in mammalian brain in relation to ketone body metabolism and regulation. G6P, Glucose 6-phosphate; Pyr, Pyruvate; TCA, tricarboxylic acid cycle; GLUT, glucose transporter.

Our patient is among the youngest reported MCT1 deficiency cases with a detailed neuroimaging assessment. Following the exclusion of other possible factors that could contribute to the abnormal brain MRI findings, a hypothesis of a possible genotype-phenotype correlation is suggested. In the brain, glucose is the most important energy substrate while lactate is an alternative one (20). The expression of MCT2 by neurons provides an optimal transporter for lactate uptake and utilization as an energy substrate in a status of increased energy demand (21). MCTs exclusively transport lactate, and change in transporters cause change in production of lactate and their utilization. MCT1 is highly localized around axons and oligodendrocytes (cells that myelinate nerve fibers) and its inactivating mutation or reduced expression leads to neuronal loss due to axonal degeneration in animal models and *in vitro* studies (22). MCT1 is expressed in human astrocytes (23) functioning in neuron survival (24). Astrocytes are the most abundantly available glial cell type in human central nervous system (CNS) (23), therefore shedding light on the importance of MCT1 in proper myelination (Figure 3). MCT1 expression is also reduced in amyotrophic lateral sclerosis patients suggesting its role in the pathogenesis (24). To our knowledge, this is the first report describing a detailed neuroimaging assessment and neurological outcome in an infant with MCT1 deficiency. Therefore, our case could potentially expand the clinical spectrum of the genetic causes of recurrent ketoacidosis. MRI findings have been described previously in other monocarboxylate deficiencies such as MCT8 (25), showing white matter disease as seen in our patient, however, there hasn't been any reported MRI on a MCT1 deficiency patients. Our study has thus been the first to provide a detailed neuroimaging assessment as a possible reference for MCT1 deficiency related neurological abnormalities. Although our case presents the first documentation of CNS alterations in patients with *SLC16A1* inactivating mutations, further functional studies will be required to understand the role of the MCT1 in key tissues such as the CNS, liver, muscle, and ketone body metabolism. However, our description of an expanded clinical spectrum sets the basis for future research to dissect the intricate mechanisms behind the clinical manifestations reported in this case. Defective role of MCT1 in mice CNS results in axon damage and neuronal loss (24), yet it remains unclear at this stage whether this is a direct effect of MCT1 loss in the CNS or a consequence of profound ketoacidosis. The threshold of blood glucose to circumvent neurological sequelae cannot be set because it is patient-specific, nevertheless, neurodevelopmental follow up is recommended in this patient. Finally, our comprehensive clinical and genetic assessment sets the grounds for endocrinologists and neurologists following up MCT1 deficiency patients, serving as a reference for proper management, follow up, and prognostic prediction.

The lactate shuttle hypothesis is supported by MCT1 presence in the sarcolemma of oxidative skeletal muscle fibers (26). Similar to oxidative skeletal muscle and heart muscle, the brain may consume lactate during high-energy demands (27). An increasing body of evidence suggests that lactate, rather than glucose is the major neuronal energy substrate for tissue surviving an ischemic insult, in addition to lactate neuroprotective function (28, 29). MCT overexpression provided additive neuroprotection, emphasizing the importance of astrocyte–neuronal lactate shuttling for preventing brain damage in conditions of high neuronal activity (30, 31). In certain circumstances lactate may enter the brain from the circulation. MCT1 appears restricted to vascular endothelium (31). During starvation and diabetic hypoglycemia the brain must in part rely on supply of monocarboxylates for production of energy. During breast-feeding, lactate and other monocarboxylates (ketone bodies) serve as important energy substrates for the infant brain (32, 33). Several lines of evidence show that MCT1 is strongly expressed on blood vessels in human cortical layers (34), suggesting an important role in human brain. In patients with medication-refractory temporal lobe epilepsy (TLE), MCT1 has been shown to have a critical role in the disease pathophysiology where MCT1 was deficient on microvessels in the epileptogenic hippocampal formation (35).

Hypothalamic neuronal circuits play an essential role in the control of feeding, glucosensing, controlling hypothalamic orexigenic, and anorexigenic neuropeptides expression and release (36–38). Interestingly, hypothalamic MCT1 expression has been associated with feeding behavior regulation; in which MCT1 inhibition results in increased food intake and weight gain in animal models (39). This phenomenon has been attributed to altered metabolic coupling between tanycytes and arcuate nucleu neurons (39). Our patient exhibited an increase in body weight, (above 97th percentile), a phenotype that could be attributed to MCT1 inhibition (39). Recent studies also suggest that increased expression of MCT1 and MCT4 play an important role in the physiological functions of adipocytes during brown and white adipogenesis (40), shedding light on possible mechanisms involved in the increased body weight seen in our patient. MCTs could, therefore, be a fundamental protein associated with nutrient sensing and regulation of food intake, ultimately constituting an interesting topic for future investigation.

## Conclusion

We report a mutation in the *SLC16A1* gene leading to recurrent ketoacidosis and hypoglycemia. Our patient is among the youngest reported MCT1 deficiency. The c.218delG variant in exon 3 in the *SLC16A1* gene is predicted to causes a frameshift and creates a premature stop codon at position 8 of the new reading frame. The variant is expected to result in loss of normal MCT1 protein function. Our case describes neurological sequelae that could be associated with MCT1 deficiency; however, functional studies are required to validate the genotype-phenotype correlation. The identification of disease causing *SLC16A1* inactivating mutations using in-depth genetic analysis could facilitate the initiation of appropriate follow up.

## Ethics Statement

Written informed consent was obtained from the parents for the publication of this case report and accompanying images. A copy of the written consent is available for review by the editor of this journal.

## Author Contributions

SA-K and KH: concept and design. SA-K, KH, and EA: writing and drafting the manuscript. SA-K: methodology. SA-K, JA, SS, FK, RH, BH, IM, EA, and KH: data analysis. KH: final approval of the manuscript.

### Conflict of Interest Statement

The authors declare that the research was conducted in the absence of any commercial or financial relationships that could be construed as a potential conflict of interest.

## References

[1] Laffel L. Ketone bodies: a review of physiology, pathophysiology and application of monitoring to diabetes. Diabetes Metab Res Rev. (1999) 15:412–26. 10.1002/(SICI)1520-7560(199911/12)15:6<412::AID-DMRR72>3.0.CO;2-8 10634967

[2] Sass JO. Inborn errors of ketogenesis and ketone body utilization. J Inherit Metab Dis. (2012) 35:23–8. 10.1007/s10545-011-9324-6 21479626

[3] Westerberg DP. Diabetic ketoacidosis: evaluation and treatment. Am Fam Physician. (2013) 87:337–46. 23547550

[4] Tildon JT Cornblath M. Succinyl-CoA: 3-ketoacid CoA-transferase deficiency. A cause for ketoacidosis in infancy. J Clin Invest. (1972) 51:493–8. 10.1172/JCI106837 4258782 PMC302154

[5] Daum RS Lamm PH Mamer OA Scriver CR. A “new” disorder of isoleucine catabolism. Lancet. (1971) 2:1289–90. 10.1016/S0140-6736(71)90605-2 4143539

[6] van Hasselt PM Ferdinandusse S Monroe GR Ruiter JP Turkenburg M Geerlings MJ. Monocarboxylate transporter 1 deficiency and ketone utilization. N Engl J Med. (2014) 371:1900–7. 10.1056/NEJMoa1407778 25390740

[7] Halestrap AP. The SLC16 gene family - structure, role and regulation in health and disease. Mol Aspects Med. (2013) 34:337–49. 10.1016/j.mam.2012.05.003 23506875

[8] Kirk P Wilson MC Heddle C Brown MH Barclay AN Halestrap AP. CD147 is tightly associated with lactate transporters MCT1 and MCT4 and facilitates their cell surface expression. EMBO J. (2000) 19:3896–904. 10.1093/emboj/19.15.3896 10921872 PMC306613

[9] Wilson MC Meredith D Fox JE Manoharan C Davies AJ Halestrap AP. Basigin (CD147) is the target for organomercurial inhibition of monocarboxylate transporter isoforms 1 and 4: the ancillary protein for the insensitive MCT2 is EMBIGIN (gp70). J Biol Chem. (2005) 280:27213–21. 10.1074/jbc.M411950200 15917240

[10] Lauritzen F de Lanerolle NC Lee TS Spencer DD Kim JH Bergersen LH. Monocarboxylate transporter 1 is deficient on microvessels in the human epileptogenic hippocampus. Neurobiol Dis. (2011) 41:577–84. 10.1016/j.nbd.2010.11.005 21081165 PMC3330827

[11] Liu B Niu L Shen MZ Gao L Wang C Li J. Decreased astroglial monocarboxylate transporter 4 expression in temporal lobe epilepsy. Mol Neurobiol. (2014) 50:327–8. 10.1007/s12035-013-8619-z 24464262

[12] Lu W Huang J Sun S Huang S Gan S Xu J Changes in lactate content and monocarboxylate transporter 2 expression in A_β25–35_-treated rat model of Alzheimer's disease. Neurol Sci. (2015) 36:871–6. 10.1007/s10072-015-2087-3 25647291

[13] Funfschilling U Supplie LM Mahad D Boretius S Saab AS Edgar J. Glycolytic oligodendrocytes maintain myelin and long-term axonal integrity. Nature. (2012) 485:517–21. 10.1038/nature11007 22622581 PMC3613737

[14] Morrison BM Lee Y Rothstein JD. Oligodendroglia: metabolic supporters of axons. Trends Cell Biol. (2013) 23:644–51. 10.1016/j.tcb.2013.07.007 23988427 PMC3842360

[15] Mosconi L. Glucose metabolism in normal aging and Alzheimer's disease: methodological and physiological considerations for PET studies. Clin Transl Imaging. (2013) 1:217–33. 10.1007/s40336-013-0026-y 24409422 PMC3881550

[16] Balasubramaniam S Lewis B Greed L Meili D Flier A Yamamoto R. Heterozygous monocarboxylate transporter 1 (MCT1, SLC16A1) deficiency as a cause of recurrent ketoacidosis. JIMD Rep. (2016) 29:33–8. 10.1007/8904_2015_519 26608392 PMC5059203

[17] Inoue K Khajavi M Ohyama T Hirabayashi S Wilson J Reggin JD. Molecular mechanism for distinct neurological phenotypes conveyed by allelic truncating mutations. Nat Genet. (2004) 36:361–9. 10.1038/ng1322 15004559

[18] Anna A Monika G. Splicing mutations in human genetic disorders: examples, detection, and confirmation. J Appl Genet. (2018) 59:253–68. 10.1007/s13353-018-0444-7 29680930 PMC6060985

[19] Genomes Project C Auton A Brooks LD Durbin RM Garrison EP Kang HM A global reference for human genetic variation. Nature. (2015) 526:68–74. 10.1038/nature15393 26432245 PMC4750478

[20] Riske L Thomas RK Baker GB Dursun SM. Lactate in the brain: an update on its relevance to brain energy, neurons, glia and panic disorder. Ther Adv Psychopharmacol. (2017) 7:85–9. 10.1177/2045125316675579 28255438 PMC5315230

[21] Perez-Escuredo J Van Hee VF Sboarina M Falces J Payen VL Pellerin L. Monocarboxylate transporters in the brain and in cancer. Biochim Biophys Acta. (2016) 1863:2481–97. 10.1016/j.bbamcr.2016.03.013 26993058 PMC4990061

[22] Zhang M Ma Z Qin H Yao Z. Monocarboxylate transporter 1 in the medial prefrontal cortex developmentally expresses in oligodendrocytes and associates with neuronal amounts. Mol Neurobiol. (2017) 54:2315–6. 10.1007/s12035-016-9820-7 26957300

[23] Ideno M Kobayashi M Sasaki S Futagi Y Narumi K Furugen A. Involvement of monocarboxylate transporter 1 (SLC16A1) in the uptake of l-lactate in human astrocytes. Life Sci. (2018) 192:110–4. 10.1016/j.lfs.2017.10.022 29154783

[24] Lee Y Morrison BM Li Y Lengacher S Farah MH Hoffman PN. Oligodendroglia metabolically support axons and contribute to neurodegeneration. Nature. (2012) 487:443–8. 10.1038/nature11314 22801498 PMC3408792

[25] Sijens PE Rodiger LA Meiners LC Lunsing RJ. 1H magnetic resonance spectroscopy in monocarboxylate transporter 8 gene deficiency. J Clin Endocrinol Metab. (2008) 93:1854–9. 10.1210/jc.2007-2441 18319316

[26] Bergersen LH Thomas M Johannsson E Waerhaug O Halestrap A Andersen K. Cross-reinnervation changes the expression patterns of the monocarboxylate transporters 1 and 4: An experimental study in slow and fast rat skeletal muscle. Neuroscience. (2006) 138:1105–13. 10.1016/j.neuroscience.2005.12.014 16446038

[27] Dalsgaard MK Quistorff B Danielsen ER Selmer C Vogelsang T Secher NH A reduced cerebral metabolic ratio in exercise reflects metabolism and not accumulation of lactate within the human brain. J Physiol. (2004) 554:571–8. 10.1113/jphysiol.2003.055053 14608005 PMC1664756

[28] Cater HL Benham CD Sundstrom LE. Neuroprotective role of monocarboxylate transport during glucose deprivation in slice cultures of rat hippocampus. J Physiol. (2001) 531:459–66. 10.1111/j.1469-7793.2001.0459i.x 11230518 PMC2278461

[29] Chih CP Roberts EL Jr. Energy substrates for neurons during neural activity: a critical review of the astrocyte-neuron lactate shuttle hypothesis. J Cereb Blood Flow Metab. (2003) 23:1263–81. 10.1097/01.WCB.0000081369.51727.6F 14600433

[30] Bliss TM Ip M Cheng E Minami M Pellerin L Magistretti P. Dual-gene, dual-cell type therapy against an excitotoxic insult by bolstering neuroenergetics. J Neurosci. (2004) 24:6202–8. 10.1523/JNEUROSCI.0805-04.2004 15240812 PMC6729663

[31] Bergersen LH. Is lactate food for neurons? Comparison of monocarboxylate transporter subtypes in brain and muscle. Neuroscience. (2007) 145:11–9. 10.1016/j.neuroscience.2006.11.062 17218064

[32] Nehlig A Boyet S Pereira de Vasconcelos, A. Autoradiographic measurement of local cerebral beta-hydroxybutyrate uptake in the rat during postnatal development. Neuroscience. (1991) 40:871–8. 10.1016/0306-4522(91)90018-J 2062444

[33] Gladden LB. Lactate metabolism: a new paradigm for the third millennium. J Physiol. (2004) 558:5–30. 10.1113/jphysiol.2003.058701 15131240 PMC1664920

[34] Chiry O Pellerin L Monnet-Tschudi F Fishbein WN Merezhinskaya N Magistretti PJ. Expression of the monocarboxylate transporter MCT1 in the adult human brain cortex. Brain Res. (2006) 1070:65–70. 10.1016/j.brainres.2005.11.064 16403470

[35] Lauritzen F Perez EL Melillo ER Roh JM Zaveri HP Lee TS. Altered expression of brain monocarboxylate transporter 1 in models of temporal lobe epilepsy. Neurobiol Dis. (2012) 45:165–76. 10.1016/j.nbd.2011.08.001 21856423 PMC3351090

[36] Schwartz MW Woods SC Porte DJr Seeley RJ Baskin DG. Central nervous system control of food intake. Nature. (2000) 404:661–71. 10.1038/35007534 10766253

[37] Burdakov D Luckman SM Verkhratsky A. Glucose-sensing neurons of the hypothalamus. Philos Trans R Soc Lond B Biol Sci. (2005) 360:2227–35. 10.1098/rstb.2005.1763 16321792 PMC1569598

[38] Lam TK Gutierrez-Juarez R Pocai A Rossetti L. Regulation of blood glucose by hypothalamic pyruvate metabolism. Science. (2005) 309:943–7. 10.1126/science.1112085 16081739

[39] Elizondo-Vega R Cortes-Campos C Barahona MJ Carril C Ordenes P Salgado M. Inhibition of hypothalamic MCT1 expression increases food intake and alters orexigenic and anorexigenic neuropeptide expression. Sci Rep. (2016) 6:33606. 10.1038/srep33606 27677351 PMC5039692

[40] Petersen C Nielsen MD Andersen ES Basse AL Isidor MS Markussen LK. MCT1 and MCT4 expression and lactate flux activity increase during white and brown adipogenesis and impact adipocyte metabolism. Sci Rep. (2017) 7:13101. 10.1038/s41598-017-13298-z 29026134 PMC5638914

